# Pseudoexfoliation syndrome in diabetic patients: transmission electron microscopy study of anterior lens epithelial cells

**DOI:** 10.22336/rjo.2021.8

**Published:** 2021

**Authors:** Fani Akritidou, Sofia Karachrysaphi, Theodora Papamitsou, Antonia Sioga

**Affiliations:** * Department of Ophthalmology, General Hospital of Serres, Serres, Greece; ** Laboratory of Histology-Embryology, Department of Medicine, School of Health Sciences, AUTH (Aristotle University of Thessaloniki), Thessaloniki, Greece

**Keywords:** pseudoexfoliation syndrome, senile cataract, diabetes mellitus, lens epithelium cells, electron microscopy

## Abstract

**Purpose:** to examine the lens epithelial cells in diabetic patients with pseudoexfoliation to ultramicroscope and to compare the findings with those of patients without diabetes mellitus (DM) and/or without pseudoexfoliation (PEX).

**Materials and Methods:** Forty patients aged 65-86 years were enrolled in the study. All patients had senile cataract and were divided into four groups of ten patients in each group. Group I: patients without pseudoexfoliation, without DM, Group II: without pseudoexfoliation, with DM, Group III: with pseudoexfoliation, without DM, Group IV (Pseudoexfoliation-Diabetic Group): with pseudoexfoliation, with DM. In all cases, part of the central portion of anterior lens capsule was removed during routine cataract surgery, and was properly prepared in order to be examined under a transmission electron microscope.

**Results:** In the control group, mainly degenerative alterations to varying extents were observed. In all groups, intracellular and extracellular oedema, multilayering, apoptosis, completely destroyed cells adjacent to normal cellswere described. In the diabetic group, alterations were more severe with respect to group I. In PEX cases, the additionalirregularity of the epithelium surface, loose intercellular connection, as well as the loose connection between cells and basement membrane were described with the presence of PEX material free and within the basement membrane. In cases with PEX and DM, degenerative alterations and PEX material were observed as well, but the epithelium was better conserved compared to the PEX group.

**Conclusion: **the observed lesions were more extended and more frequent in the pseudoexfoliation group, followed by the diabetic group. The pseudoexfoliation-diabetic group presented less intense modifications raising questions about the interaction of these different diseases.

**Abbreviations:** DM = Diabetes Mellitus, PEX = Pseudoexfoliation, PXM = Pseudoexfoliative Material, AD = Alzheimer disease, TGF-β1 = Transforming Growth Factor beta 1, WHO = World Health Organization, LEC = Lens Epithelium Cells, BM = Basement Membrane, CM = Cytoplasmic Membrane

## Introduction

Pseudoexfoliation syndrome (PEX) is an age-related systemic microfibrillopathy characterized by the progressive deposition of whitish-gray extracellular material, the pseudoexfoliative material (PXM), in various intraocular and extraocular tissues. Indeed, PXMcan also be found in many different organs such as the heart, liver, kidneys, lungs, cerebral meninges, vessel walls and skin and, according to many studies, it could be associated with numerous systemic abnormalities including transient ischemic attacks, hypertension, angina, myocardial infarction, cerebrovascular and cardiovascular disease, aortic aneurysm, renal artery stenosis, Alzheimer disease (AD) and hearing loss [**1**-**2**].

PEX is commonly observed in people older than 50 years of age. In fact, different epidemiological studies concluded that, in all populations, occurrence is negligible in the middle-aged population (49–54 years), and the prevalence of the disease increases markedly with age [**3**,**4**]. Although it has been reported in all population types and races, there is a significant variation of incidence and prevalence of PEX in different countries and regions. PEX principally affects northern Europeans and especially Scandinavians [**3**,**4**]. Even though the exact etiology of PEX still remains unclear, it is well known that multiple factors, including genetic, geographic, and environmental factors are implicated in its pathogenesis, explaining the different prevalence of the disease worldwide [**4**].

The PEX-fibrotic matrix process is a stress-induced elastosis [**5**]. Indeed, according to several studies, cellular stress conditions (oxidative, ischemia/hypoxia) play a crucial role in the pathobiology of PEX [**6**,**7**]. PEX-fibrosis is characterized by an excessive production and abnormal cross-linking of elastic microfibrils into fibrillar PEX aggregates. This fibrotic process could be triggered by many different conditions such as increased expression and activity of transforming growth factor beta 1 (TGF-β1) [**8**], imbalance between matrix metalloproteinases and their tissue inhibitors [**9**,**10**], increased oxidative stress [**11**,**12**], anterior chamber hypoxia [**13**], low-grade inflammatory processes [**2**,**5**]. Furthermore, recent genetic studies identified LOXL1 variants highly correlated to PEX as they are present in almost 100% of PEX patients worldwide. LOXL1 is a cross-linking enzyme important for elastic fiber formation and stabilization in the extracellular matrix. Thus, LOXL1 risk variants are considered the most important risk factor for manifestation of the PEX phenotype [**5**]. The site of production of this material is unclear, but PXMcan potentially originate from the iris, lens epithelium, ciliary body, or the trabecular meshwork [**14**,**15**]. 

On the other hand, Diabetes Mellitus (DM) is a very common pathology among aged people. According to World Health Organization (WHO), the prevalence of DM is high and rising in every country [**16**]. DM is a complex metabolic disease characterized by chronic hyperglycemia, resulting from defects in insulin secretion, insulin action, or both, and involves a wide range of genetic and environmental factors [**17**]. It is well known that the influence of this metabolic disorder on the ocular structures can lead to several conditions such as diabetic retinopathy, diabetic papillopathy, glaucoma, cataract, and ocular surface diseases. Diabetic ocular complications represent one of the major causes of blindness even though early detection and timely treatment may ameliorate visual prognosis [**18**]. More specifically, cataract is one of the major causes of visual impairment in diabetic patients [**19**] and cataract formation in diabetics seems to be related to the levels and duration of hyperglycemia. In addition, DM seems to precipitate senile lens opacity [**20**]. Indeed, diabetic patients develop cataract at an earlier age and 2-5 times more frequently compared to the general population [**21**,**22**]. Although the exact pathogenesis is not fully understood, there are several different mechanisms that contribute to diabetic-cataract formation [**18**], including increased osmotic stress caused by activation of the polyol pathway [**23**], non-enzymatic glycation of lens proteins [**24**,**25**] and increased oxidative stress [**26**,**27**]. 

Regarding the lens epithelium, Struck et al. (1997) found that there is a significantly lower mean cell density in type-II diabetics [**28**] and Kim et al. concluded that the apoptosis in lens epithelium cells (LEC) is increased in cataract patients with DM [**29**]. In vivo and in vitro experiments triggered the induction of apoptosis and the attenuated cell growth, resulting in decreased lens epithelial cell density in diabetic patients [**30**-**32**].

The aim of this paper was to study and confront the changes of the anterior capsular bag and the LEC in cataract patients with pseudoexfoliation and diabetes mellitus and to investigate if there is an interaction of these conditions on this tissue.

## Material and methods

Forty patients were enrolled in this study. All patients were adults aged between 65 and 86 years and they were all programmed for routine cataract surgery. Patients with non-senile cataract and patients with type I diabetes were excluded from this study. Only patients with type 2 diabetics with disease duration of over ten years were included in this study.

Before the operation, the nature of the study was thoroughly explained and patients signed informed consent forms. The Ethical Committee of the Aristotle University of Thessaloniki approved the study.

All patients had senile cataract and were divided into four groups. Each group consisted of 10 patients:

Group I – Control Group: without pseudoexfoliation, without Diabetes Mellitus;

Group II: without pseudoexfoliation, with Diabetes Mellitus;

Group III: with pseudoexfoliation, without Diabetes Mellitus;

Group IV: with pseudoexfoliation, with Diabetes Mellitus.

In all cases, samples of anterior capsular bag were collected intraoperatively during uneventful cataract surgery. During the routine cataract surgery, more specific during capsulorhexis, part of the central portion of anterior lens capsule was removed in order to gain access and remove the crystal lens. For this study, this material was collected in order to be examined ultramicroscopically.

All types of samples were processed along identical protocols.

For this study, the tissues were immediately placed in fixative (3% glutaraldehyde) solution for 2 hours. Then, the tissues were postfixed in 1% osmium tetroxide for 1 hour and after they were washed in distilled water, they stained en bloc with 2% aqueous uranyl acetate for 16-20 h at 4°C in dark. After dehydration of the tissues through an increasing density of alcohols, they were embedded in Epon (epoxy resin, Sigma, Japan) embedding medium. Finally, thin sections (60-150 nm) were cut with an ultramicrotome (LEICA EM UC6), stained with Reynold’s solution, and were examined under a TEM JEOL-1011 in 80 KV transmission electron microscope.

All statistical analysis was performed using SPSS v.25.0 (IBM Corp., Armonk, NY, USA).

## Results

The study included 40 eyes of 40 participantsaged between 65 and 86, with a mean age of 77.4 ± 5.5 years (mean ± standard deviation) and 57.5% were women.

The demographic data of the patients included in each group are shown in **Table 1**.

**Tabel 1:** Demographic data

Group	Description	Number of participants	Gender M:F	Age (mean ± standard deviation)
I	Control Group	10	6:4	77.9 ± 5.3
II	DM Group	10	4:6	75.6 ± 6.8
III	PEX Group	10	4:6	77.1 ± 5.3
IV	DM-PEX Group	10	3:7	78.9 ± 4.5

In the ultrastructural study, the anterior lens epithelial cells revealed changes between the four groups.

**Fig. 1:**
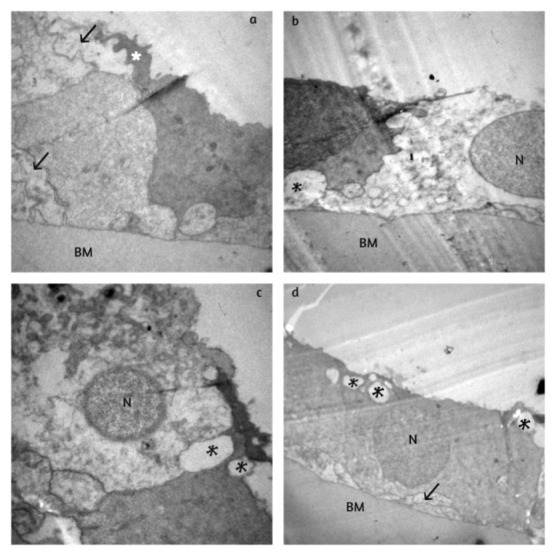
**(a)** Group I: Degenerative alterations with features of intracellular oedema and damaged cells with pycnotic nuclei (N); **(b,c)** vacuoles (black asterisks), cytoplasmatic processes and multilayering (black arrows); **(d)** tight epithelium attachment to basement membrane (BM)

In the control group/ group I (**Fig. 1**), we observed degenerative alterations to varying extents. Intracellular vacuoles of various sizes, oedema with cytosolic dilution and apoptotic nuclei were observed. Moreover, there were cytoplasmic processes that coveredthe neighboring cells and so we observed a multilayer epithelium. The attachment of the basement membrane (BM) was generally smooth and tight.

**Fig. 2:**
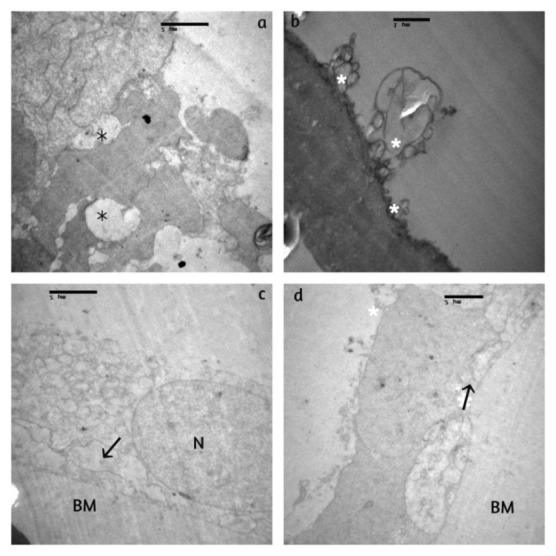
Group II: Complete destruction of cells with ruptures of the cell membrane adjacent to normal cells, extensive diffuse intracellular oedema **(a,c)**, intercellular vacuoles (black asterisks), apoptotic nuclei (N), **(b,d)** cytoplasmatic processes on both sides of the epithelium and multilayering (black arrows) round-shaped formations (white asterisks)

The alterations in group II with DM (**Fig. 2**) were serious. Intracellularly, there were more vacuoles, enlarged endoplasmic reticulum, swollen mitochondria, and more apoptotic nuclei. Large intracellular spaces and many damaged cells were noticed. Many cells were connected with the basement membrane, but there were some that completely detached from it. More cytoplasmic processes, not only to the upper side of the epithelium but also towards the underside of it, were noticed and so the epithelium was multilayered.

**Fig. 3:**
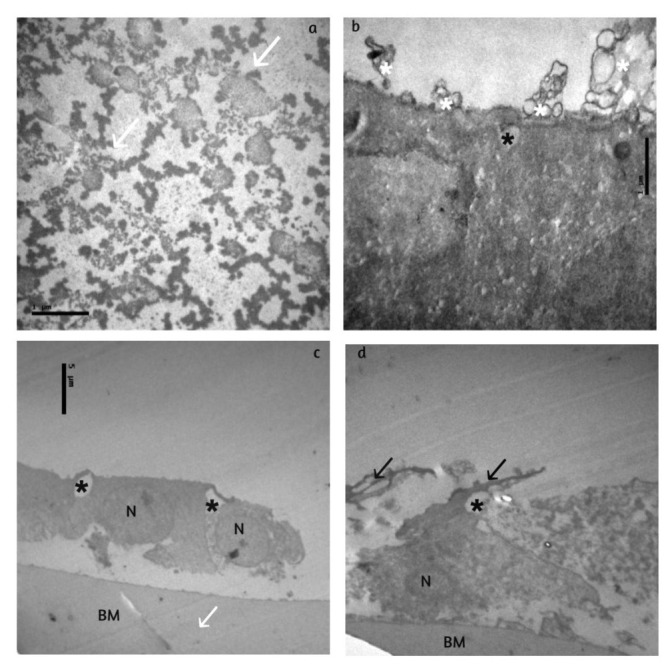
Group III: there are many totally damaged cells **(c,d)**, intracellular vacuoles (black asterisks), apoptotic nuclei (N), oedema, increased intercellular spaces, cytoplasmatic processes to the upper and underside of the epithelium with multilayering (black arrows), rough and irregular surface of the epithelium, round-shaped formations (white asterisks) **(b)**, abnormal electrodense material (white arrow) in the ground substance **(a)** and within the basement membrane (BM). The attachment to the BM and the intercellular connection is very loose and very often a total detachment of the cells occurs **(c,d)**

The alterations in group III with PEX (**Fig. 3**) were more severe. More pronounced cytoplasmic processes of different sizeswere observed to the upper and underside of the epithelium (multilayering). In some areas, the lens epithelial cellswere totally lost. Many times, the thickness of cytoplasmic membrane (CM) was larger and the surface of the epithelium was rough with some round-shaped formations, probably from the cell membrane. Abnormal deposits of fine randomly arranged, electron dense material was noticed in the ground substance or in the basement membrane. Many totally damaged cells and many intracellularvacuoles (small, large, lamellar), swollen mitochondria, enlarged rough endoplasmic reticulum, many apoptotic nuclei, edema and increased intercellular spaces were observed.

**Fig. 4:**
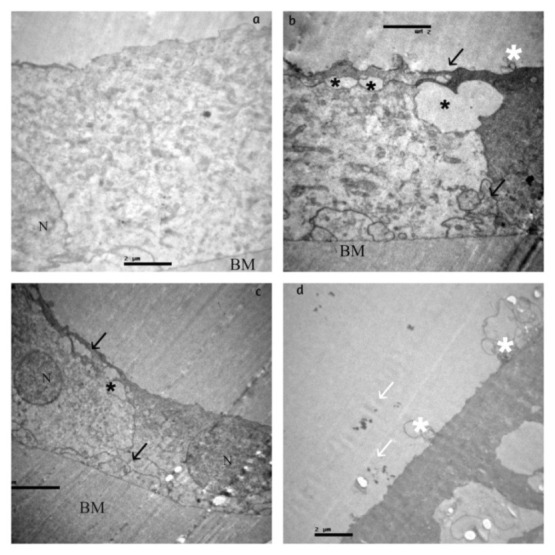
Group IV: Degenerative lesions of the lens epithelium of various severity are described with apoptotic nuclei (N), diffuse intracellular oedema **(a,b)**, degenerative vacuoles of different size (black asterisks), cytoplasmatic processes covering adjacent cells and aspect of multilayering (black arrows), irregularity of the apical surface of the epithelium **(c). (d)** Free microgranular, electron-dense material (white arrow) is observed above the apical cellular membrane of the lens epithelium and round-shaped formations (white asterisks) at the outer cellular membrane surface. Intercellular connection and attachment to the basement membrane (BM) is well preserved

The alterations in group IVwith PEX and DM (**Fig. 4**) were similar with the ones in group III. The extracellular material was abundant in the upper side of the epithelium and in the basement membrane. The intracellular alterations were more intensive, but there were fewer round-shaped formations of the outer surfaces of the cell membrane.

## Discussion

Histologically, the crystal lens is composed of the lens capsule, the lens epithelium, and the lens fiber-cortex. The anterior lens capsule is a modified transparent basement membrane attached to the lens epithelial cells. It is secreted by the lens epithelial cells and it is the thickest basement membrane in the human body. Anterior lens epithelial cells are flattened cuboidal hexagonal cells, tightly packed in a single layer with very little intercellular space and they contain a round nucleus with a few apically distributed organelles [**33**,**34**].

The lens capsule and the lens epithelium, acting as a regulating barrier between the aqueous humor and the lens fibers, allow the passive and active exchange of ions, nutrients, and liquid. They play a crucial role in maintaining appropriate lens osmolarity in order to preserve lens transparency. When the transport processes, morphology or biochemistry of the lens capsule and the epithelium are disturbed, lens transparency could be compromised leading to cataract formation[**33**-**34**].

The present study was directed towards investigating transmission electron microscopic findings of the anterior lens capsule epithelium incataract patients with PEX and DM and comparing them with those from age matched controls. 

In age-related cataract, the capsule, and the epithelium present typical agingmorphologicalchanges, such as altered hexagonal cellular arrays and changes in the endoplasmic reticulum, the Golgi apparatus, and the mitochondria [**34**,**35**]. According to different studies, the transmission electron microscopy findings of senile cataract are mainly apoptotic and degenerative changes, with the presence of intercellular and intracellular vacuoles influencing the appearance of both the nucleus and the entire cell, cytoplasmic processes over adjacent cells and multilayering of LECs [**36**-**42**]. According to Charakidas’ et al. (2005), the epithelial apoptosis in cataract does not result in significant cell density decrease because epithelial gaps could be replaced by cell proliferation at the germinative zone of the anterior lens capsule [**41**]. More severe and intense changes are present in cases of mature cataract. In these cases, epithelial cells could be severely damaged with loss of cell membrane integrity and complete destruction of the cell [**43**]. So, in senile cataract, the severity and the extension of these lesions could vary depending on the patients’ age and the maturity of the cataract and, in many cases, normal appearing epithelial cells were found adjacent to severely damaged cells [**35**,**42**,**44**]. The findings of our study were in accordance with the above-described observations, as degenerative alterations to varying extents were described in all samples of group I.

As far as diabetic cataract is concerned, despite its morphological similarity, lenses with cataracts in diabetics have lower epithelial cell density than those in healthy individuals [**34**,**45**-**47**]. Indeed, the anterior capsular bag and the LEC in DM presents all the above-described morphological features of aging [**28**,**34**] with higher rate of apoptosis [**48**,**49**]. Erol et al. suggested that diabetes mellitus affects foremost the organelles of anterior lens capsule epithelium by promoting structural abnormalities of organelles including mitochondrial crystalysis, dilation of the endoplasmic reticulum cistern and apoptotic dense nucleus [**34**]. In our study, the alterations of the LECs in group II with DM were indeed like the ones in the control group but more severe and extended. There were more vacuoles and larger intracellular spaces. Also, many damaged cells were noticed and many cells were completely detached from the basement membrane.

Regardingpseudoexfoliation syndrome, the incidence of cataract formation is increased in patients with PΕX [**50**]. In addition, in cases of uniocular PΕX, the affected eye often demonstrates the more advanced cataract [**51**-**53**]. Although the cataract formation could simply be a consequence of the advanced age of PΕX-patients as no causal link has been proven [**2**,**50**], Oltuluet al. suggested that the pathophysiological mechanism for the higher rate of cataracts in PEX cases is the increased apoptosis in LECs, along with the ocular ischemia hypothesis [**54**].

The first to perform light microscopy on an eye with PEX was Archimede Busacca and later the advent of the electron microscope allowed a more detailed analysis of this tissue [**55**,**56**]. Degenerative alterations, with intra- and inter-cellular vacuoles, cellular edema, apoptotic changes, irregularity of the epithelium surface, cytoplasmic processes and abnormal electrodense material free and within the basement membrane are the main features of the LECs described in literature [**42**,**55**,**56**]. Overall, the severe alterations described above are comparable to the lesions observed in our samples.

Pseudoexfoliation syndrome in diabetic patients has not been fully studied yet. Although the coexistence of aging, diabetes mellitus and pseudoexfoliation is expected to cause more severe alterations, the abnormalities of the LECs described in our study were less intense. The general impression was that the whole epithelium was more compound in comparison with group II and III, although we expected more extended and severe modifications. This was probably because of the interaction of these conditions, which is still unclear. At this point, we must underline that the present study was limited by the small size of our sample. Further and more extended research is needed in order to extract more secure conclusions for group IV.

## Conclusions

Comparing the findings between the four groups, the observed lesions were more extended and more frequently described in the exfoliation group, followed by the diabetic group. Surprisingly, the pseudoexfoliation-diabetic group presented less intense modifications, which raised questions about the interaction of these different diseases. From this interesting observation, interesting questions about the interaction of these conditions could be raised. The small size of the sample of this study and the fact that pseudoexfoliation syndrome in diabetic patients is not yet fully explored in literature leads to the conclusion that further research is needed to justify these results.

**Conflict of Interest**

No potential conflict of interest is reported by the authors.

**Informed Consent and Human and Animal Rights statements**

Informed consent has been obtained from all individuals included in this study.

**Authorization for the use of human subjects**

Ethical approval: The research related to human use complies with all the relevant national regulations, institutional policies, is in accordance with the tenets of the Helsinki Declaration, and has been approved by the Ethics Committee of General Hospital of Serres, Serres, Greece.

**Acknowledgements**

The data that support the findings of this study are available on request from the corresponding author, Fani Akritidou. The data are not publicly available due to privacy restrictions.

**Sources of Funding**

The authors received no specific funding for this work.

**Disclosures**

None.
